# Impact of Providing Feed and/or Water on Performance, Physiology, and Behavior of Weaned Pigs during a 32-h Transport

**DOI:** 10.3390/ani6050031

**Published:** 2016-05-03

**Authors:** Arlene Garcia, Mhairi Sutherland, Glenna Pirner, Guilherme Picinin, Matthew May, Brittany Backus, John McGlone

**Affiliations:** 1 Department of Animal and Food Sciences, Texas Tech University, Lubbock, TX 79409, USA; agarciam@crk.umn.edu (A.G.); glenna.pirner@ttu.edu (G.P.); Guilherme.picinin@ttu.edu (G.P.); maymattmd@gmail.com (M.M.); brittany.backus@ttu.edu (B.B.); 2 Department of Agricultural and Natural Resources, University of Minnesota, Crookston, MN 56716, USA; 3 Ruaura Research Centre, AgResearch Ltd., Hamilton 3214, New Zealand; mhairi.sutherland@agresearch.co.nz

**Keywords:** animal welfare, behavior, transport, weaned pigs

## Abstract

**Simple Summary:**

Transportation has the potential to negatively affect the health and welfare of weaned pigs, especially those already experiencing weaning stress. Piglets were transported for 32 h, with and without feed and water, and measures of performance, physiology, and behavior were taken to assess piglet welfare. Transportation negatively impacted body weight, Neutrophil to Lymphocyte Ratio (N:L), and post-transport body weight gain, indicating that not providing water during transport can negatively impact the well-being of recently weaned pigs. Provision of water may aid in reducing stress during long distance transport and improve the animals’ well-being.

**Abstract:**

Transportation at weaning is a complex stressor made up of many factors, including withdrawal from feed and water, which can potentially negatively affect the health and welfare of pigs, especially those already experiencing weaning stress. The objective of this study was to evaluate the effect of weaning and extended transport durations (up to 32 h), with and without the provision of feed and/or water, on pig welfare. Treatment groups included: pigs neither weaned nor transported, control (CON); weaned pigs transported and provided with feed and water (T+); weaned pigs transported without feed and water (T−); weaned pigs transported with only feed (T+F); and weaned pigs transported with only water provided (TRAN+W). The effect of transport (with and without feed and/or water) on weaned pigs was assessed using behavior, performance, and physiology. After a 32-h transport period, pigs transported without water lost markedly more weight than those transported with water (*p* < 0.01). Furthermore, the neutrophil to lymphocyte ratio was markedly higher in male pigs transported without water (*p* < 0.05). Overall, transportation had a negative effect on pig well-being, especially when water was not provided.

## 1. Introduction

Road transport of weaned piglets from rearing to growing-finishing farms is a common practice in the swine industry [1]. Transportation is a complex stressor made up of many factors including fluctuating temperatures, stocking density, withdrawal from feed and water, mixing with unfamiliar pigs and motion [2]. Therefore, transportation has the potential to affect the health and welfare of pigs, especially in pigs already experiencing weaning stress. In Europe, animals are often transported between different countries. However, current European Union regulations limit transport of animals to 8 h, although pigs can be transported for up to 24 h as long as water is provided [1,3]. Currently in the United States, animals can be transported consecutively for 28 h without the need to be off-loaded, or provided with feed, water, or rest [4].

This 28 h law was enacted in 1873 and applied to rail transportation of cattle, sheep, swine, and other animals and was later amended to include transportation by express or common carriers involving confinement in a “vehicle or vessel” [4]. It is important to provide scientific validation for recommendations to enhance the welfare of animals. However, the 28 h law was not based on science or current common transport methods for weaned pigs. Therefore, research is needed to develop science-based recommendations for transporting weaned pigs that is relevant to modern conditions.

Transportation of pigs at weaning is becoming more common in the U.S. and is an essential aspect of modern swine production [1,5,6]. Weaned piglets are typically transported from short to very long distances from rearing to growing-finishing farms typically to reduce vertical transfer of disease, to enhance production, and overall farm efficacy [6] and the duration of transport can vary from short to very long distances. Transport of weaned pigs has many common factors with transport of finishing pigs, including mixing, fasting, temperature fluctuations, vibration, novelty and noise [7]. Similar to studies in finishing pigs, transport has been shown to cause behavioral and physiological changes indicative of stress in weaned pigs [6]. However, there is limited information regarding the effect of transport duration and the associated effect of feed and water deprivation on the welfare of weaned pigs. Weaning itself is a stressful event for piglets. Piglets are separated from the sow, their diet changes from milk to solid feed, they are mixed with unfamiliar pigs, are exposed to a novel environment, and all these factors can contribute to stress [8]. Weaning and its associated stressors can lead to a period of underfeeding, growth retardation and metabolic changes in pigs until they adapt [8,9]. In addition, weaning can affect the immune system, performance and behavior of pigs [10,11]. Therefore, pigs already experiencing stress due to weaning maybe more vulnerable to additional stressors, such as transport.

Furthermore, there is limited information regarding the effect of transport duration and the associated effect of feed and water deprivation on the welfare of weaned pigs. The current study is a continuation of a previous study conducted by this laboratory, where we concluded that feed and water should be provided to weaned pigs if transport will be over 24 h, based on measures of body weight, physiology, and behavior [12]. Hence, it is necessary for the industry to understand the impact of transport on the welfare of weaned pigs so that standards can be put into place to reduce any detrimental effects associated with transporting pigs at weaning.

The objective of this study was to evaluate the effect of weaning and extended transport durations (up to 32 h), with and without feed and/or water, on pig welfare using measures based on behavior, performance, and physiology. Transport times up to 32 h were assessed to provide scientifically based information to determine if the 28 h law in the USA is appropriate for weaned pigs.

## 2. Experimental Section

Pigs were PIC USA (Hendersonville, TN, USA) genetics using the Camborough-22 sow line. All animals were fed a diet to meet or exceed National Research Council (NRC) nutrient requirements. Feed and water were provided *ad libitum* or according to treatment. All animal procedures were approved by the Texas Tech University Animal Care and Use Committee (13040-06).

Pigs were ear notched, tail docked, needle teeth clipped, and males were surgically castrated at 3 d of age. Pigs were ear tagged 3 d prior to the beginning of the study for identification purposes. At 18 to 22 d of age (the average weaning age of pigs on commercial swine farms in the U.S.), pigs were randomly assigned to one of five treatment groups. Pigs were selected by date of birth ±2 d and weight (in order to get similar weights among groups). Each treatment group weighed 6.0 ± 0.58 kg when selected for the study. Both gilts and barrows were evenly represented in each treatment group (2 sexes × 5 treatments = 10 pigs × 6 replications = 60 pigs). Pigs were randomly selected for one of five treatment groups:

(1) Not weaned or transported: pigs remained with the sow (CON); *n* = 12.
(2) Weaned, transported, but had access to feed and water: pigs were removed from the sow and transported for 32 h in pens that had feed and water (T+); *n* = 12.
(3) Weaned, transported but had no access to feed and water: pigs were removed from the sow and transport for 32 h in pens that did not have feed and water (T−); *n* = 12.
(4) Weaned, transported with access to feed: pigs were removed from the sow and transported for 32 h in pens with access to feed only (T+F); *n* = 12.
(5) Weaned and transported with access to water: pigs were removed from the sow and transport for 32 h in pens with access to water only (T+W); *n* = 12.

The study was conducted in the United States during early fall (avoiding temperatures that were too cold or too hot, to prevent thermal stress). The study began at 07:00 h and ended by 16:00 h the following day. At weaning, blood samples were taken from each individual control (CON) pig and pigs were either left in the farrowing pen with the sow or placed in one of the four treatment groups. All pigs were blood sampled by jugular venipuncture, weighed, checked for lesions or lameness at 0 h and every 8 h thereafter up to 32 h. Feed and/or water intake was measured every 8 h.

For treatment groups two to five, two pigs (one barrow and one gilt) from the same litter were randomly placed in pens (0.76 m × 0.76 m) with wood shavings, approximately 10 cm in depth, in a goose-neck trailer (6 m × 2 m). Space allowance was determined based on the Transport Quality Assurance [13] guidelines for transporting pigs (0.19 m^2^/head) and the additional space (0.14 m × 0.14 m) was added to provide space for feed and water. HOBO**^®^** (Onset Computer Corporation, Bourne, MA, USA) data loggers were placed on each experimental pen to record temperature and humidity in the trailer at the level of the pig. The average temperature in the trailer was 25.5 °C and relative humidity inside the trailer ranged from 17.9% to 83.5%.

Pigs were transported for 32 h on main highways for the majority of the trip, which were sealed, predominantly straight, and involved minimal stopping/acceleration or inclines/declines. The 32 h of transport were broken down into 8-h phases, in which the truck/trailer would return to the original farm site for sampling purposes. Sampling took approximately 15 to 25 min and involved weighing, bleeding, and assessing pigs for injuries. Feed (starting at 2.5 kg) and water (starting at 5 L) consumption were determined by weighing the feed containers and measuring volume in water jugs every 8 h for the 32 h treatment period. Travel time continued during the sampling periods, so that sampling took place at 0, 8, 16, 24 and 32 h. At the end of the 32-h transport period, all pigs including CON pigs were placed in weaning pens (1.5 m × 1.5 m) with the same pen mate to avoid further stress caused by mixing of unfamiliar pigs.

To assess piglet welfare, measures of performance (body weights, health, and injury), physiology, and behavior (during transport, at the farm, and post-transport) were measured.

*Performance*: Piglets were weighed by placing them in a plastic tub that was set on top of a digital bench scale (OHAUS, Melrose, MA, USA). The pigs were weighed before (0 h), during (at 8, 16, and 24 h), immediately after (32 h), and at 7- and 14-d post study period. Percent weight change was calculated. Before, during, and after transport each pig was examined for injuries, wounds, abscesses, and lameness.

*Physiology*: Pigs were placed on their backs in a V-trough with their forelegs and hind legs manually restrained by trained personnel for blood collection, which took on average 2 to 3 min. Two milliliters of blood was collected through jugular venipuncture into vacutainers (BD Vacutainers**^®^**, Becton, Dickinson and Company, Franklin Lakes, NJ, USA) containing 5.4 mg of K2 EDTA and an additional 2 mL were collected into vacationers (BD Vacutainers**^®^**, Becton, Dickinson and Company, Franklin Lakes, NJ, USA) without additives, for blood analysis. Four mL in total were collected from every piglet every 8 h for 32 h. Whole blood was examined for hematocrit (HCT), and neutrophil to lymphocyte ratio (N:L) within 1 h after collection. Blood samples were centrifuged and plasma and serum were collected and frozen until further analysis for cortisol concentrations. Cortisol concentrations were analyzed using an enzyme immunoassay kit (Enzo**^®^** Life Sciences, Farmingdale, NY, USA). The resulting intra- and inter-assay coefficient of variation (CV) were 6% and 10.4% respectively. The sensitivity of the assay was 1 pg/mL.

*Behavior*: Piglet behavior was recorded for all treatment groups during and 24 h after the study. Lying, standing, sitting, drinking, and eating behaviors were recorded according to Garcia *et al.* [12] (Table 1). To record behaviors of all transported treatment groups during transport, wild life cameras (Moultrie Products, Alabaster, AL, USA) were placed across from each experimental pen. The behavior of four pigs (2 pens) were recorded per frame and 10 min scan samples were done for each individual pen. The cameras were motion activated and instantaneous shots were taken upon activation (movement of the pigs). Digital video recorders (DVRs) (Supercircuits VSS Series, Supercircuits**^®^**, Austin, TX, USA) were used to record behaviors of CON pigs in farrowing pens. Post-transport pigs were penned with the same pen mate they had during transport. Video was also taken for 24 h post-transport for all groups and quantified to examine potential treatment effects. Videos from DVRs were analyzed in 10 min scan samples in 2 h intervals.

*Statistical Analysis*: The study was a Complete Randomized Design. The pen was the experimental unit, consisting of one gilt and one barrow. All data were tested for homogenous variances and departures from normal distribution using the univariate procedure of SAS version 9.1 SAS (SAS, 2010 SAS Inst., Inc., Cary, NC, USA). All data were subjected to analysis of variance using the general linear model procedure of SAS. The statistical model included the main fixed effects of sex, treatment, pen, time, and all possible interactions. Pen within treatment was used as the model’s error term. An F-protected Least Significant Difference test was also used within SAS. Behavior was recorded using 10 min scan samples in 2-h intervals.

## 3. Results and Discussion

### 3.1. Performance

#### Body Weight Loss

The treatment by time interaction was significant for percent change in body weights (*p* < 0.01). All treatments groups were different from CON starting at 8 h of transport (Table 2). All transported treatment groups displayed similar losses in body weight from 0 to 24 h. However, by 32 h of transport the T− and T+F groups had lost more weight compared to T+, T+W and the CON group (*p* < 0.05).

These data suggest that water withdrawal during transport has a greater effect on weight loss than feed withdrawal. Weight loss was more than likely due to dehydration, and therefore water withdraw may be more important than feed withdrawal and the effect increases with duration of transport. Similarly, Garcia *et al.* [12] found that pigs transported and/or weaned without the provision of feed and water lost significantly more weight than control pigs and pigs that were weaned and transported and provided with feed and water. These findings are also consistent with results from studies investigating slaughter weight pigs that have been transported and fasted for 25 to 48 h [6,7,8,14,15,16,17,18,19,20,21]. However in the present study, with the exception of CON pigs, percent change in body weight was similar among treatment groups prior to 32 h. These results are in general similar to the findings of Garcia *et al.* [12], who found differences in body weight loss between pigs transported with and without the provision of feed and water within 24 h of transport.

### 3.2. Physiology

#### 3.2.1. Neutrophil to Lymphocyte Ratio

**Figure 1:**
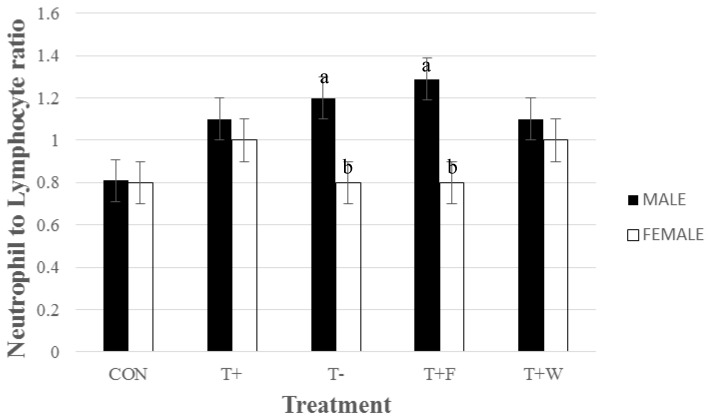
Least Squares means ± SEM for neutrophil to lymphocyte ratio (N:L) for sex by treatment interaction over 32 h for pigs weaned and transported or neither weaned nor transported (*p* < 0.01). Treatments: neither weaned nor transported (CON), weaned and transported; with feed and water provided (T+), without feed and water provided (T−), only feed provided (T+F), and with only water provided (T+W). *n* = 12 pigs/treatment. Superscripts without a common letter within treatment group differ at *p* < 0.05.

N:L was not significantly different among treatment groups. However, a significant treatment by sex interaction for N:L was observed (*p* < 0.01). T+F males had higher N:L than T+F females (*p* < 0.05; Figure 1). T− males also exhibited higher N:L than T− females (*p* < 0.05). Whereas, the N:L was similar among male and female pigs transported with water. The current findings suggest that male pigs that did not have access to water or feed during transport may have experienced more stress than females in response to the same stressors. Differences in the stress response between genders could potentially be explained by the differences in their early life experiences. For example, male pigs experience a greater degree of stress at processing due to surgical castration than females. The stress caused by castration may predispose male pigs to be more responsive to future stressors, such as human handling, due to conditioning effects causing pigs to associate handling with acute pain [22]. Literature is limited on whether painful processes, such as castration in pigs, applied early in life increases pain perception later on. It has been documented that circumcision in young boys is associated with greater pain perception at vaccination than uncircumcised boys [23]. Therefore, the negative experience of castration may cause an increase in stress during handling, blood sampling, and transportation compared to females. However, further investigation is needed to understand the relationship between pig gender, castration and the stress response associated with transport (with and without feed/water).

Transport and weaning individually have been shown to influence the immune response, performance, physiology, and behavior of young pigs [10,11,24]. Weaning alone, which involves transitioning from milk to solid feed, fasting, and being mixed with unfamiliar pigs, can lead to an increase in the N:L. Studies on the effect of fasting on the N:L have been reported [7,8,18,19,20,21,23,25,26,27] but there are limited studies that describe the effect of providing feed and/or water during transport on the physiology of recently weaned pigs.

#### 3.2.2. Hematocrit

HCT values were not different among the treatment groups (*p* > 0.05). The values for the CON, T+, T−, T+F, and T+W groups were 28.70% ± 1.61%, 30.32% ± 1.70%, 29.23% ± 1.72%, 28.29% ± 2.5%, and 28.85% ± 1.91%, respectively. Previous evaluation of the effects of 6, 12, 18, 24, or 30 h of transport in gilts showed that HCT values did not increase in response to transport [20], but other measure of dehydration such as albumin and total protein concentrations were elevated. In a similar study by Garcia *et al.* 2015 the effect of long distance transport in weaned pigs for up to 32 h showed that both albumin and HCT values did not increase regardless of whether water and feed were provided. These finding are in contrast with previous reports where hematocrit values have been reported to increase during transport of varying durations [1,20,28].

#### 3.2.3. Cortisol

Cortisol concentrations were not different among the treatment groups (*p* > 0.05). The values for the CON, T+, T−, T+F, and T+W groups were 20.32 ± 8.90, 44.72 ± 11.55, 29.09 ± 8.78, 41.97 ± 8.93 and 28.89 ± 8.90 pg/mL, respectively. Cortisol concentrations have been reported to increase in response to transport stress [20,21]. Averos *et al.* [1] did not find any cortisol differences in weaned pigs being transported regardless of short (0.6 h) or long distance transport (8.3 h). In the current study, just as in other reports, it seems that stress associated with handling may have been greater than any potential treatment effects [12,24].

### 3.3. Behavior

#### 3.3.1. During Transport

During the 32 h transport period, CON pigs spent 58% of their time lying. T+, T−, T+F, and T+W pigs spent 66%, 67%, 73%, and 54% of their time lying, respectively. Lying behavior varied among treatments over time, but T+F displayed more differences than the other groups (Table 3). T+F spent more time lying than CON pigs during 2 h, 6 h, 16 h, and 32 h of transport (*p* < 0.05). T− displayed lying behavior differences compared to the CON at 14 h, 18 h, and 32 h; T+ at 10 h and 32 h; and T+W at 22 h and 32 h of transport (*p* < 0.05).

T+ spent more time lying than T+W at 10 h, 18 h, 20 h, and 22 h of transport, but were similar to the CON except at 10 h of transport, where T+ spent more time lying than both T+W and CON (*p* < 0.05). T+W spent less time lying than T+ but were similar to the CON, except at 22 h, where T+W spent less time lying than both the T+ and CON (*p* < 0.05). T− spent similar amounts of time lying to T+F, except at 2 h of transport, where both T− and CON spent less time lying than T+F (*p* < 0.05). All transported treatment groups spent more time lying compared to CON pigs at 32 h (*p* < 0.05). A diurnal cycle, although difficult to interpret can still be identified from 18 h to 22 h.

Transported pigs may spend more time lying due to the unstable conditions caused by transport. Garcia *et al.* [12] similarly found that recently weaned pigs, transported with or without the provision of feed and water, spent more time lying than control (non-transported) pigs. Alternatively, increased lying behavior may be indicative of increased welfare, as these pigs may be less restless or habituated to their environment. The current study has similar results to other studies where weaned pigs have been previously reported to spend most of their time lying and standing during transport, 75.6% and 21.6%, respectively [7]. It has also been reported that high levels of resting after weaning is common in pigs, which could be associated with the increased lying behavior during transport [27].

CON pigs spent 16% of their time standing during the 32-h study period. T+, T−, T+F, and T+W pigs spent 20%, 25%, 18%, and 21% of their time standing, respectively.

Sitting behavior was not observed in CON pigs but was observed in all other treatment groups. Both T+ and T+F pigs spent 3% of their time sitting during transport while T+W and T− spent 2% of their time sitting.

In addition, as transport time increases, pigs tend to spend more time lying [8,28]. Sitting is a behavior that has been identified as a potential stress indicator [5], and has been reported to be more common during the first 12 h of transport (2.8%) than in the second 12 h (0.3%) [8]. Increased standing or sitting behavior has also been suggested as an indication of competition for space or instability due to too much space [29]. However, neither was a problem in the present study due to the space allowance used. Increased resting and a decrease in sitting behavior later in transport may indicate that piglets have become habituated to some of the elements of transport [7].

It is important to also consider that the type of vehicle used to transport weaning pigs, as they may have a different effect on transport behaviors. In the current study, a 6 m × 2 m livestock trailer was used, which is different to what may typically be used in a commercial setting (in the USA straight deck and potbelly livestock trailers may range from 14.7 m to 16.2 m long and 2.6 m wide). There may be differences between large and small trailers with respect to movement related behavior of the vehicle, just like there are differences in morbidity and mortality associated with large commercial trailers [30]. The main aim of this study was to evaluate the effect of feed/water withdrawal, so, in this instance, the dimensions of the trailer used in the study are less critical. However, the construction of the vehicle and driving style is still very important. Driving style may overrule all other precautions to protect piglet welfare. Additionally, raising the standards of the type of vehicle used in the U.S. to transport piglets, such as special suspensions, heating/air conditioning, feeding and watering for long distance transport like is currently used in Europe can help improve weaned pig welfare. Based on the current findings, larger studies on commercial farms using more commercially applicable trailers are needed to substantiate/confirm these results.

#### 3.3.2. Feed and Water Consumption during Transport

CON pigs were observed to spend 23% of their time nursing during the 32 h study period. T+F pigs spent 1% of their time eating while T+ pigs spent 2% of their time eating. There was no significant treatment effect for feed consumption, both T+ and T+F groups ate similar amounts of feed, 0.19 ± 0.77 kg and 0.29 ± 0.09 kg, respectively. However, a treatment by time interaction was observed for pigs provided with feed (*p* < 0.05; Table 4). T+F pigs spent more time eating at 10 h of transport compared to T+ pigs. Pigs consumed more feed at 24 h of transport (*p* < 0.05) compared to 8, 16, and 32 h of transport. Feed consumption over the 32 h transport period starting at 8 h was 0.06 kg, 0.06 kg, 0.64 kg, and 0.20 kg, respectively. Drinking behaviors were not observed during transport, or may have been missed due to video being scanned in 10 min intervals. Occasionally equipment failure occurred resulting in periods of missed data collection, which may also have been a reason for the low observed occurrence of feeding and drinking behavior. There was no significant treatment effect for water consumption, both the T+ and T+W drank similar amounts of water, 0.58 ± 0.09 L and 0.42 ± 0.09 L, respectively. Overall, drinking behavior was more prevalent at 16 h of transport compared to 8 and 24 h (*p* < 0.05), but drinking behavior was similar among treatments at 32 h of transport (*p* > 0.05). The increase in water consumption at 16 h and the increase consumption of feed at 24 h may have allowed the pigs to maintain their weight over the rest of the transport period. However, it is difficult to know if water was actually consumed. There may have been some spillage, which is normally seen even when animals are not being transported.

#### 3.3.3. Behavior Post-Treatment

CON pigs were weaned after the 32 h study period. Upon being weaned, CON pigs spent 72% of their time lying. T+ and T− pigs both spent 73% of their time lying after transport, while T+F and T+W spent 77% and 67% of their time lying, respectively.

CON pigs spent 23% of their time standing after the 32 h study period. T+ and T+W both spent 13% of their time standing while T−, T+F, and spent 19% and 17% of their time standing after transport, respectively.

Both CON and T+ pigs spent 2% of their time eating after the 32 h study period. T+W, T− and T+F pigs spent 13%, 5%, and 4% of their time eating after transport, respectively. Drinking behaviors were not observed in CON pigs post-weaning. T+, T−, T+F, and T+W pigs spent 1%, 3%, 2%, and 5% of their time drinking, respectively.

T+W pigs appeared to spend less time lying and standing and more time eating and drinking than the other transported animals. However, the difference between these behaviors was not significant. Pigs spend more time resting, feeding, and drinking during the 3 d after transport than the non-transported control animals [8]. This is likely due to exhaustion, dehydration, and hunger. In future studies, it may advantageous to increase the sampling time of the videos to be able to detect behaviors that are exhibited at low frequencies, such as eating and drinking. It may also be beneficial to observe behavior for longer than 24-h post-transport.

#### 3.3.4. Feed and Water Consumption after Transport

Feed and water intake was recorded at 7 and 14 d post-treatment, but no differences were found among treatment groups (*p* > 0.05). Similar results in feed and water intake post-treatment were reported by Garcia *et al.* [12]. This may indicate that weaned and transported pigs, regardless of what treatment group they were, they ate and drank similarly over time.

#### 3.3.5. Post-Transport Body Weights

Post-transport body weights were recorded at 7 and 14 d. Body weight was similar among all treatment groups at 7 and 14 d post-transport (*p* > 0.05). However, a sex by treatment interaction for post-transport body weight was observed (*p* < 0.01). Sex differences were found within the CON and the T+ treatment groups. CON males had a lower average weight compared to females, 7.77 ± 0.28 kg and 8.57 ± 0.28 kg, respectively. T+ males had a higher average weight than females, 8.34 ± 0.40 kg and 7.16 ± 0.27 kg. Male pigs have been reported to be heavier at birth than females [30], but sex differences are typically not seen at this early of an age. There were several pigs in the study that could be considered poor-doers, piglets below weaning weights after 7 d post-transport. Poor-doers are piglets that are still not meeting their physiological requirements after 7 d post-transport and have been documented to be an indicator of poor welfare [7].

## 4. Conclusions

Weaned pigs are exposed to similar transport stresses as finishing pigs, in conjunction to also concurrently experiencing weaning stress, which is the most stressful event in an animal’s life. However, literature pertaining to the effect of transportation on the welfare of weaned pigs is limited [6,18,19,20,21,31,32,33,34,35,36,37]. Transport is an added stressor in a pig’s life that many times cannot be avoided. The use of a multidisciplinary approach (e.g., performance, physiology, behavior, morbidity, and mortality) to investigate implications of transporting and different aspects of transport (e.g., duration, space allowance) on pigs at weaning should be further studied [6].

In the current study, pigs weaned and transported with or without feed and water, lost significantly more body weight than CON animals by 8 h of transport. In addition, animals not provided with water lost significantly more weight than those provided with water by 32 h of transport. This suggests that for recently weaned pigs, there is no need to provide feed during transportation, but provision of water may aid in reducing stress during transport over 24 h. Furthermore, providing water may help weaned pigs habituate to their current environment. In addition, physiological and behavioral changes were apparent after weaning and during transport. N:L increased significantly in males that were not provided with water compared to females, possibly suggesting that castrated males may experience acute stress at a higher level than females. The overall appreciation of the results points towards the usefulness of providing water during long distance transport, and this is already applied in practice in some countries.

Further research is needed to determine the long-term effects of long distance transport on weaned pigs in relation to performance, physiology, and behavior. Studies investigating the provision of water in relation to stocking density, season, and transport duration would be beneficial to the pork industry. Using other measures of welfare in addition to the ones already established may help give better insight into the physiological changes associated with long distance transport of weaned pigs, and the benefits of providing water during transport on the animals’ welfare.

## Figures and Tables

**Table 1:** Ethogram, description of behaviors during and post-treatment.

Behavior	Definition
*Lying*	Animal is recumbent, flat on its side, or ventrally
*Standing*	Animal is upright on all fours, legs are extended
*Sitting*	Resting on the caudal part of the body with the forelimbs extended
*Drinking*	Animal placing its mouth on the water nipple and consuming water
*Eating*/*Nursing*	Animal placing its mouth in the feed container and consuming feed or latching on to the sows‘ nipple and suckling

**Table 2:** Least Squares means ±0.09 for percent change in body weight over 32 h for pigs weaned and transported or neither weaned nor transported (*p* < 0.01). Treatments: neither weaned nor transported (CON), weaned and transported; with feed and water provided (T+), without feed and water provided (T−), only feed provided (T+F), and with only water provided (T+W). *n* = 12 pigs/treatment.

Time, h	Treatments				
	CON	T+	T−	T+F	T+W
0	0	0	0	0	0
8	6.09 ^**a**^	−2.43 ^**b**^	−2.48 ^**b**^	−2.45 ^**b**^	−2.86 ^**b**^
16	9.04 ^**a**^	−3.80 ^**b**^	−4.65 ^**b**^	−4.58 ^**b**^	−3.92 ^**b**^
24	11.24 ^**a**^	−4.91 ^**b**^	−6.11 ^**b**^	−5.83 ^**b**^	−4.78 ^**b**^
32	13.80 ^**a**^	−5.66 ^**b**^	−7.44 ^**c**^	−7.51 ^**c**^	−5.51 ^**b**^

**^a^**^–**c**^ Means with different superscripts within each time point differ at *p* < 0.05.

**Table 3:** Least squares means ±2.13 for treatment by time interaction for lying behavior over 32 h for pigs weaned and transported or neither weaned nor transported (*p* < 0.05). Treatments: neither weaned nor transported (CON), weaned and transported; with feed and water provided (T+), without feed and water provided (T−), only feed provided (T+F), and with only water provided (T+W). *n* = 12 pigs/treatment.

Time, h	Treatments				
	CON	T+	T−	T+F	T+W
2	8.67 ^**a**^	12.67 ^**a**^	13.33 ^**a**^	14.67 ^**b**^	9.17 ^**a**^
4	12.17 ^**a,b**^	9.83 ^**a**^	16.0 ^**b**^	19.17 ^**b**^	11.50 ^**a,b**^
6	9.67 ^**a**^	12.00 ^**a**^	15.00 ^**a,b**^	18.00 ^**b**^	9.50 ^**a**^
8	11.33 ^**a,b**^	9.67 ^**a,b**^	13.00 ^**a,b**^	15.17 ^**a**^	9.17 ^**b**^
10	12.33 ^**a,c**^	19.50 ^**b**^	15.50 ^**a**^	16.00 ^**a**^	8.67 ^**c**^
12	13.17	14.00	11.67	15.00	12.67
14	15.67 ^**a**^	12.50 ^**a,b**^	9.50 ^**b**^	13.67 ^**a,b**^	14.83 ^**a,b**^
16	10.33 ^**a**^	14.33 ^**a,b**^	14.17 ^**a,b**^	19.50 ^**b**^	12.67 ^**a**^
18	16.67 ^**a,c**^	19.67 ^**a**^	22.50 ^**b**^	20.00 ^**a,b**^	11.50 ^**c**^
20	17.17 ^**a,b**^	22.00 ^**a**^	21.50 ^**a**^	18.50 ^**a,b**^	14.17 ^**b**^
22	20.83 ^**a**^	21.00 ^**a**^	22.00 ^**a**^	18.00 ^**a,b**^	13.17 ^**b**^
24	13.00	13.67	15.50	16.00	11.50
26	13.50	15.50	11.50	14.67	13.33
28	17.00	19.50	17.00	19.83	14.83
30	19.00	17.67	18.83	19.50	20.50
32	9.26 ^**a**^	18.33 ^**b**^	21.67 ^**b**^	23.83 ^**b**^	19.00 ^**b**^

**^a^**^–**c**^ Means with different superscripts within each time point differ at *p* < 0.05.

**Table 4:** Least squares means ±0.54 for treatment by time interaction for eating behavior over 32 h for pigs weaned and transported (*p* < 0.05). Treatments: pigs with feed and water (T+) or feed only (T+F). *n* = 12 pigs/treatment.

Time, h	Treatments		
	CON	T+	T+F
2	5.83 ^**a**^	0.33 ^**b**^	0.67 ^**b**^
4	6.50 ^**a**^	0.17 ^**b**^	0.00 ^**b**^
6	8.50 ^**a**^	0.5 ^**b**^	0.00 ^**b**^
8	5.67 ^**a**^	0.17 ^**b**^	0.17 ^**b**^
10	7.83 ^**a**^	0.50 ^**b**^	2.00 ^**c**^
12	8.00 ^**a**^	0.50 ^**b**^	0.67 ^**b**^
14	7.17 ^**a**^	1.17 ^**b**^	0.17 ^**b**^
16	8.17 ^**a**^	0.17 ^**b**^	0.00 ^**b**^
18	6.17 ^**a**^	0.50 ^**b**^	0.00 ^**b**^
20	5.50 ^**a**^	0.00 ^**b**^	0.00 ^**b**^
22	1.67 ^**a**^	0.00 ^**b**^	0.00 ^**b**^
24	4.50 ^**a**^	1.00 ^**b**^	0.00 ^**b**^
26	4.17 ^**a**^	1.00 ^**b**^	0.33 ^**b**^
28	4.33 ^**a**^	0.33 ^**b**^	0.67 ^**b**^
30	2.67 ^**a**^	0.50 ^**b**^	0.50 ^**b**^
32	0.78 ^**a**^	0.00 ^**a**^	0.00 ^**a**^

**^a^**^–**c**^ Means with different superscripts within each time point differ at *p* < 0.05.
